# Development of a pre-hospital emergencies protocol for the management of suicidal patients in Iran

**DOI:** 10.1186/s12873-021-00437-z

**Published:** 2021-04-14

**Authors:** Fatemeh Shirzad, Shakiba Gholamzad, Maisam Shafiee, Seyed Vahid Shariat

**Affiliations:** 1 Spiritual Health Research Center, Iran University of Medical Sciences, Tehran, Iran; 2 Student Research Committee, Iran University of Medical Sciences, Tehran, Iran; 3 Department of Clinical Psychology, School of Behavioral Science and Mental Health (Tehran Institute of Psychiatry), Iran University of Medical Science, Tehran, Iran; 4 Mental Health Research Center, Psychological Health research institute (PHRI), Iran University of Medical Science, Tehran, Iran; 5 School of Behavioral Science and Mental Health (Tehran Institute of Psychiatry), Iran University of Medical Science, Tehran, Iran

**Keywords:** Pre-hospital emergency, Protocol, Suicide, Management, Iran

## Abstract

**Background:**

Suicide is a painful consequence of many psychiatric disorders and one of the most frequent psychiatric emergencies. Generally, pre-hospital technician is the first person in the treatment chain who attends the situation; hence, his/her sound clinical judgment and professional behavior can play an important role in preventing or stopping the suicide process. We tried to develop a concise, evidence-based, and step-by-step guide for dealing with a suicidal patient, which could be quickly reviewed by technicians before confronting a suicide situation.

**Method:**

We reviewed the literature for suicide management plans and protocols, to extract the evidence-based interventions and instructions for dealing with a suicide situation. Then, we discussed the extracted material in an expert panel, and developed the initial version of the protocol considering the local socio-cultural issues and available facilities. Subsequently, we reviewed the protocol in a meeting with pre-hospital technicians and emergency physicians, to receive their feedback and address any possible executive problems. Finally, we revised the protocol to its final version considering the feedbacks.

**Results:**

The basic principles of dealing with a suicidal patient are similar to other psychiatric emergencies and include: Patient Safety; Patient evaluation and diagnosis; and Patient (behavioral and pharmacological) management. However, specific considerations should be taken into account and special arrangements are necessary for suicidal patients. Whether the patient has attempted suicide or not, would guide the management to one of the two major paths. In addition, the needs of the family should be considered.

**Conclusion:**

A locally adapted protocol considering existing facilities in the emergency system and cultural issues in Iranian society is provided for pre-hospital emergency technicians.

## Background

Suicide is an intentional act to end one’s own life. Wish to kill oneself may remain limited to thought (suicidal ideation) or turn into action (suicidal attempt) and on some occasions result in death (committed suicide) [1]. Nearly 800,000 people commit suicide each year in the world and still many more attempt suicide [2]. Suicide is the second leading cause of death between the ages of 15–29 in the world [3]. Suicide is a serious public health issue and has several risk factors including previous suicide attempts, unemployment, substance use, and being affected by at least one psychiatric disorder. Major depressive disorder and substance use disorder have been the most common mental health problems associated with suicide [4].

The prevalence of suicide attempts in Iran has increased in the last two decades [5]. The prevalence of suicide attempts in Iran varies from 16.8 per 100 thousand people in a year in south of Iran to 117.8 per 100 thousand people in a year in north of Iran [6]. The highest rate of completed suicide in Islamic Republic of Iran is 12.9 per 100,000 which is related to Western Provinces, including Ilam, Lorestan, Hamedan, Kurdestan and Kermanshah. One of the methods of suicide in this area is self-immolation (about 27% of all suicides). About 80% of self-immolation cases are committed by women. Self-immolation is more common in the first years of marriage when there are many emotional problems and conflicts and low coping skills between couples. Studies attribute issues such as the decline in the Human Development Index, cultural factors and negative attitudes toward women, and the reduction of suicidal stigma [7, 8]. The lowest rate is 2.8 per 100,000 related Central Provinces including Isfahan, Yazd, Semnan and Qom [9].

According to Iranian Social Welfare Organization (ISWO), the social emergency of ISWO has taken preventive action in 8400 suicide attempts through mobile teams (> 3000 cases), social service bases (242 cases), crisis intervention centers (1300 cases), and contact with social emergency hotline (3400 cases) in past year [10].

Pre-hospital emergency has also been involved as a first line contact in suicide situations in the country and has played an important role in this case [11]. They are the most widespread medical emergency facility in throughout the county and in most cases are the first line contact with possible suicidal patients and should provide interventions based on their prioritization, clinical judgment, and decision-making skills. Therefore, they are generally trained for the skills of how to deal with a suicide situation [12].

In critical cases such as suicide attempts, emergency technician should transfer the patient to a suitable medical center, after appropriate crisis management. Inadequate skills and mastery of emergency technicians in these conditions can lead to serious injury and even death in a person who threatens to commit suicide. Furthermore, poor decision-making ability in critical situations, increases psychological stress on emergency technician [13–16]. Therefore, different countries try to provide coherent and comprehensive clinical programs for suicide management so that the emergency technician can use them in the face of a patient threatening to commit suicide. In the United States, Dr. Betz described the important principles of suicidal patient management (patient safety, attention to physical issues, and suicide risk assessment) in his article, given the limited time and the likelihood of impending injury [17]. Dr. Christopher reviewed existing protocols for managing suicide in a pre-hospital emergency room in Washington to evaluate and integrate them to achieve a coordinated and comprehensive protocol in the future [11]. In Brazil, 15 psychiatrists and psychologists, after a systematic review of sources and articles, provided guidelines and recommendations for the Brazilian Psychiatric Association for the management of patients with suicide [18]. In Iran, there are cultural and religious issues that affect the relationship between the patient and the technician such as the need for covering, maintaining a minimum distance with patient, not touching an opposite sex patient. There are also barriers that have been identified in previous studies Ordinary people (who have no information about vital and psychological first aid) are crowded at the scene, which can complicate the decision for the technician [19].The purpose of this study was to develop a Compatible with culture, concise, evidence-based, and step-by-step guide for dealing with a suicidal patient, which could be quickly reviewed by technicians before confronting a suicide situation.

## Method

### Review of literature

This study was performed in two stages with the mixed method. We used the literature review to access the relevant resources. First, we searched PubMed, Scopus, and PsycINFO databases for the combination of the following keywords: 1)"Suicide” (OR) “suicide prevention” (OR) “suicide risk factors” (OR) “self-harm” (OR) “suicidal patients”. 2) “Management” (OR) “protocol” (OR) “pre-hospital care. Then, we combined the two searches using AND operator. We limited the search date to the results published in the recent 10 years (from June 1, 2010 to June 1, 2020). Additionally, we reviewed the chapters related to suicide management in psychiatric textbooks.

In the next step, we reviewed the abstract of the retrieved articles and selected 30 relevant articles. Subsequently, we used the Cochrane checklist to assess the full text of the articles and select suitable articles for further analysis. In this way, 20 articles were selected for the next stage (Table 1). We used these 20 articles and the textbooks for data extraction.

**Table 1:** Characteristics of the twenty studies selected for the review phase of the study and their main results

Tile	Author/s	Time	Design	Target population	objective	Main points/ result	Sample size
Adolescents’ Engagement with Crisis Hotline Risk-management Services: A Report from the Emergency Department Screen for Teen Suicide Risk (ED-STARS) Study	Busby D., et al [20]	2019	RCT	Adolescents	examines the feasibility of a risk-management protocol for adolescent research participants at risk for suicide	majority of youth share information with counselor about one or more coping strategies, Engagement did not vary by gender, race, age, ethnicity, or clinical characteristics	234 people
Assessment and Management of Patients at Risk for Suicide: Synopsis of the 2019 U.S. Department of Veterans Affairs and U.S. Department of Defense Clinical Practice Guidelines	Sall J., et al [21]	2019	Practice Guideline	Patients at Risk for Suicide	assessing and managing patients who are at risk for suicide	This synopsis summarizes the key recommendations of the guideline related to screening and evaluation, risk management and treatment, and other management methods	–
Caring for Suicidal Patients	Brent D., et al [22]	2019	Clinical Review	Suicidal Patients	Recommendations to clinicians	there are 7 evidence-based key elements for effectively treating suicidal patients	–
Management of patients with an advance decision and suicidal behavior: A systematic review	Nowland R., et al [23]	2019	systematic review	patients with an advance decision and suicidal behavior	synthesis existing literature on the management of advance decisions and suicidal behavior	Recommendations for practice and supervision for clinicians may help to reduce the variation in clinical practice	–
Assessing psychiatric safety in suicidal emergency department patients	Brenner J., et al [24]	2019	review	suicidal emergency department patients	review assessment tools (Screening tools and psychiatric consultation) and consider ethical issues	Ethical and legal considerations are important, Suicidal patients as well as those who are intoxicated or psychotic may lack capacity and require involuntary treatment	–
The Role of Emergency Medical Services Providers in Preventing Suicide	Suicide Prevention Resource Center [25]	2013	review	Emergency Medical Services Providers	examine the Role of Emergency Medical Services Providers in Preventing Suicide	review all dimensions Role of Emergency Medical Services Providers in Preventing Suicide	–
Framework for Suicide Risk Assessment and Management	NSW Health [26]	2004	review	NSW Health Staff	Provide Framework for Suicide Risk Assessment and Management for NSW Health Staff	Examine Components of a comprehensive suicide risk assessment	–
Assessment and management of agitation in Psychiatry: Expert consensus	Garriga M., et al [27]	2016	Expert consensus	Patient with agitation	A thorough and balanced review plus an expert consensus for guide assessment and treatment decisions	emphasis the importance of identifying any possible medical cause, considering physical restraint as a last resort strategy, Regarding pharmacological treatment, oral or inhaled formulations should be preferred in mildly agitated patients, Intravenous treatments should be avoided	–
Emergency Responders Management of Patients Who May Have Attempted Suicide	Lipton L.,et al [28]	2005	interview	EMS professionals	Assembling of the related topics to the management of Patients Who May Have Attempted Suicide	EMS professionals’ role in managing patients at risk for suicide or who have attempted suicide is critical, people who have made an attempt or who are threatening a lethal attempt are quite at risk for another attempt	–
Suicide Risk Assessment and Management in the Psychiatry Emergency Service: Psychiatric Provider Experience and Perceptions	Chunduri S., et al [29]	2019	Expert panel, thematic analysis	psychiatric providers working in the PES of a large urban teaching hospital	explore suicide risk identification and flow of patients with differing suicide risk through the Psychiatric Emergency Service (PES) to their clinical dispositions	screening tools cannot replace clinical judgment, the existing electronic health record is not efficient and sufficiently informative, competing demands challenge PES psychiatrists, post-discharge patient outcome data are needed	15 psychiatric providers
Suicide Risk Assessment and Management	NSW Department Of Health [30]	2004	Review	NSW Health Staff	Framework for Suicide Risk Assessment and Management for NSW Health Staff	Examine framework for Suicide Risk Assessment and Management	–
Managing suicide risk in primary care: Practice recommendations for behavioral health consultants	Jacobs D., et al [31]	2004	review	psychiatrist, nurses	1. Develop processes for accurate psychiatric assessment of patients with suicidal behaviors in various clinical settings. 2. Select appropriate treatment settings for patients with suicidal behaviors based on risk assessment. 3. Identify effective pharmacologic and psychosocial interventions for patients with suicidal behaviors	Past suicide attempts are among the most significant risk factors for suicide, and recent attempts are of particular importance.	
Treating non suicidal self-injury (NSSI) in adolescents: consensus based German guidelines.	Plener P., et al [32]	2016	Expert panel	psychiatrist, clinician	Prepare a clinical guide for treatment NSSI	Core elements of psychotherapy should be provided in treatment of NSSI. A specific psychopharmacological therapy of NSSI cannot be recommended	
The pharmacological management of acute behavioral disturbance: Data from a clinical audit conducted in UK mental health services.	Paton c., et al [33]	2019	Data analysis	psychiatrist, clinician	To describe the medication regimens used to manage episodes of acute behavioral disturbance in routine clinical care in mental health services in the UK	Behavioral disturbance involves violence towards others, a combination of parenteral haloperidol and lorazepam is most often used rather recommended. The initial attempt to manage acutely disturbed behavior with parenteral medication may fail to achieve a calming effect in up to one in four episodes	
Suicidal patients presenting to secondary and tertiary emergency departments and referral to a psychiatrist: A population-based descriptive study from Japan	Chihara I., et al [34]	2018	population-based descriptive	Suicidal patients in secondary and tertiary emergency departments in Tochigi prefecture in Japan	describe the characteristics of suicidal patients and the referral rates to a psychiatrist overall and by type of facility	professional organizations suggest that suicidal patients are seen by a psychiatrist, many were not, especially at secondary emergency departments	81 suicidal patient
Emergency department management of suicidal adolescents	Kennedy S., et al [35]	2004	Review	adolescents with suicidal ideation or attempts	Review the literature for recommendations for the management of adolescents with suicidal ideation or attempts	Hospitalization is recommended for adolescents who have attempted suicide and cannot be adequately monitored and kept safe outside of an inpatient setting. Discharge home can include adolescents who are not actively suicidal, do not have access to lethal methods, and have a supervising adult who can closely monitor their behavior. A mental health evaluation is recommended before emergency department discharge whenever feasible	–
Emergency Department (ED) Screening for Suicide and Mental Health Risk	Babeva K., et al [36]	2016	Review	low-income and minority youths who often lack a regular source of care	review the context in which ED screenings occur, available tools and strategies, and evidence for the effectiveness of tested approaches	developed brief therapeutic assessment approaches have demonstrated success in improving rates of follow-up care after discharge from the EDs. there is some data supporting clinical benefits when youths receive evidence-based outpatient follow-up care. ED screening combined with effective follow-up, may provide one strategy for improving mental health and reducing health disparities	–
Suicide Prevention in an Emergency Department Population: The ED-SAFE Study	Miller I., et al [37]	2017	Clinical trial	Adults with a recent suicide attempt or ideation	Determine whether an ED-initiated intervention reduces subsequent suicidal behavior	Among at-risk patients in the ED, a combination of brief interventions administered both during and after the ED visit decreased post-ED suicidal behavior	1376 people
Improving Suicide Risk Screening and Detection in the Emergency Department	Boudreaux E., at al [38]	2016	RCT	People with intentional self-harm ideation/behavior	Examine whether universal suicide risk screening is feasible and effective at improving suicide risk detection in the emergency department	Universal suicide risk screening in the ED was feasible and led to a nearly twofold increase in risk detection. identification of risk is the first and necessary step for preventing suicide	236,791 ED visit records
Multicomponent Intervention for Patients Admitted to an Emergency Unit for Suicide Attempt: An Exploratory Study	Brovelli S., et al [39]	2017	Clinical trial	suicide attempters	Evaluation the feasibility and acceptability of a multicomponent intervention for suicide attempters admitted to an emergency units	Joint crisis plan and meetings will have to be modified in order to improve their feasibility and acceptability, especially among first-time attempters	107 people

The extracted findings were then categorized according to the stage of intervention. The first stage is similar to the stages for other acute psychiatric patients, which are described in detail in the “pre-hospital emergency protocol for mental disorders in Iran” [16]. Here, we report the special and additional points related to suicidal patients including: ensuring safety in suicidal patients; evaluation and history taking in suicidal patients; behavioral and pharmacological management in suicidal patients; important points to reduce life-threatening risks in suicidal patients.

### Protocol development

Two psychiatrists categorized the extracted points and prepared the first draft of the protocol. Then, an expert panel was held including the following people: 1. an assistant professor of psychiatry with clinical and research experience with acute psychiatric patients, 2. a professor of psychiatry with years of experience in the emergency department of a training psychiatric hospital, 3. a professor of psychiatry with years of experience in the field of suicide and president of the National Suicide Prevention Association, 4. a professor of forensic medicine with related clinical experience, 5. a clinical psychologist with years of clinical experience with non-pharmacological therapies of patients, 6. a specialist in emergency medicine working in the country’s emergency organization with years of experience of working with emergency technicians, 7. a general practitioner working in the country’s emergency organization with years of clinical experience, 8. a clinical psychologist working in Social Emergency of Welfare and Rehabilitation Organization.

In the panel, participants were asked to comment about the framework and steps of the protocol and also to answer the following questions:

1. To what extent is this step necessary for a suicidal patient?
2. To what extent is this item understandable and transparent to an emergency technician?
3. Is this item inclusive (considering both patient’s physical and mental needs)?
4. Considering the existing facilities and conditions in our society, is this a plausible step to perform?
5. Has enough attention been paid to the patient’s safety at this stage?
6. Has enough attention been paid to the safety of the emergency technician?
7. Has enough attention been paid to the safety of the patient’s relatives and those present at the scene?
8. Considering the laws of the country, does this item have legal legitimacy for the technician?

Each question was scored on a Likert scale from very low to very high (1–5). Experts answered all of these questions for each protocol item. And the scores of 8 experts participating in the meeting for each item were added together. Finally, items with a total score of 27 and above (more than two- thirds of the total score) were selected for the protocol, and a score of 13 or less (less than one- third of the total score) was removed, and the items with the average score of 14–26 were discussed again and revised to a final agreed upon format for the protocol.

To check the clarity and comprehensibility of the revised protocol and the feasibility of its implementation in pre-hospital emergency setting, we held two sessions with two different group of people to receive feedback about the protocol and consider possible executive problems. The first session included emergency technicians and technical and operational deputy of one of the Emergency Centers of Tehran (about 50 people). The second session was held with general practitioners (dispatch) of operations in the pre-hospital emergency department. Prior to the start of both sessions, informed consent was obtained from the participants.

Then, we revised the protocol another time considering the feedbacks of the sessions and finalized the protocol in a two-page format that could be easily used in the ambulance as a quick review of important measures in dealing with a suicidal patient before confronting him/her.

## Result

The general measures that should be considered in the management of suicidal patients are not much different from those of other psychiatric emergencies, as reported in “pre-hospital emergency protocol for mental disorders in Iran” [16] and include the following three levels. For those patients who have attempted suicide, the technician should skip the first two levels and begin with management. For those who are threatening to attempt suicide, the technician should begin with level one.

1. Basic and safety tips (patient, technician and those at the scene):

The first stage (primary action) are including a) pre-scene assessments of site security, escape routes, and safe locations in the event of violence from the patient, b) assessment of the patient’s access to weapons and equipment that could threaten his/her own life, technicians or attendees [30], c) Assessment for risk and need for back up and the presence of police, which includes anticipating their entrance method and avoidance of entering the place alone, d) using family capacities to provide security [30] and e) Assessment of risk factors for violence and predicting it Symptoms of imminent aggression) [16]. In addition, the police should be contacted from the beginning. Dispersal of people present at the scene should be done immediately, as their presence may play a provocative role in attempting suicide [22]. Furthermore, when the suicide threat is made with dangerous measures such as firearms, explosives or chemicals, the presence of crowd can be quite dangerous [30].

2. Important points for patient assessments:

It includes history taking from patient and his family as well as assessing patient’s physical condition, especially life-threatening cases, and examining the possibility of a medical origin for symptoms, and psychological assessments. The suicide threat should not preclude the consideration of medical and physical causes for psychiatric symptoms.

Regarding suicide management, patients are divided into two groups:

1. Those who has not attempted suicide:

In these patients, the following were suggested:

A) Try to build a relationship with patient using respect and empathy. Avoid threats, humiliation and judgmental behavior.

In these situations, generally both patient and family members are agitated and emotionally unstable. Therefore, it is important for technician to remain calm and patient in dealing with them [40]. Many patients may refuse to cooperate because of fear of hospitalization. Thence, the use of behavioral techniques can be effective (Table 2).

B) Evaluate vital signs and ensure the stability of patient’s medical condition
C) Evaluate patient’s history and directly ask about intent for another suicidal attempt [31].
D) Risk assessment of suicide: Items listed in the table below increase the risk of suicide (Table 3).

**Table 2:** Behavioral recommendations in dealing with a suicidal patient

• Introduce yourself, explain your role and the help that you can provide • Talk in a calm and confident tone • Invite the patient to talk about his/her problem • Pay attention to patient’s cultural and religious conditions (such as the need for covering, maintaining a minimum distance with patient, not touching an opposite sex patient if Muslim) • Reduce external stimuli and existing noise as much as possible • Ask the police to stay out of patient’s sight (if the presence of police makes patient irritable and restless) • Do not leave patient alone • Do not make false promises to calm patient • Take any threat of suicide attempt seriously • Encourage patient to provide information of relatives who can help

**Table 3:** Some of suicide risk factors

• Existence or history of depression and aggression • Recent failure or loss • High-risk mental conditions such as (hopelessness, severe restlessness and agitation, or extreme guilt feeling) • Being a housewife or unemployed • Being exposed to domestic violence • History of previous suicide attempt or self-harm (especially recent ones and using dangerous methods) • Recent consumption or intoxication with alcohol or other substances • Access to guns or other deadly methods • Lack of a strong social or family support network • Instability in family or existence of family conflicts • Having a suicide plan or preparing suicide means	

Verbal threat to commit suicide and history of previous suicide attempt, especially with dangerous methods, and a history of aggression are more important risk factors [41, 42].

In addition to the items listed in Table 3, If the patient threatening to commit suicide is from the northern or western provinces of the country (especially in young married women with a history of marital problems, a history of psychiatric illnesses, especially major depression and post-traumatic stress disorder, a past history of self-immolation) Be aware of the possibility of self-immolation and examine the evidence [7, 8].

Although the assessment of suicide risk factors can help in providing better care and a more accurate evaluation of the patient, according to the current pre-hospital emergency guidelines in Iran, all of these patients, regardless of the assessed risk, should be transferred to medical centers. If the patient or his family refuse to go to hospital, the technician should contact the attending physician and ask for advice.

The following points are of great importance, provided that the patients becomes agitated and behaves in a threatening way and seems to be potentially dangerous for oneself or others:

1. Pay attention to people at risk present on the scene and maintain their safety [43].
2. Use the aggression management protocol including behavioral and drug managements (full description in the protocol for dealing with an acute psychiatric patient).
3. Transfer the patient to the hospital.

E) Collect empty containers of toxins, alcohol, and drugs, for being delivered to the hospital emergency department; even if the patient and his family claim that s/he has not attempted suicide. It is medically important to examine evidence to the contrary and can prevent legal consequences for the technician [44].
F) Documentation of the findings: The emergency technician should document all of the relevant findings and evidence to prevent further legal problems (such as what condition the patient was in at the time of his presence in terms of consciousness, vital signs, scene conditions, etc.) [44].

2. In a person who has attempted suicide:

In a person who has attempted suicide, immediate attention to the patient’s medical risks is a priority. Therefore, the measures include the following:

- A. Measuring vital signs:

The first step is to consider the patient’s immediate medical needs, initial assessment (Airway - Breathing - Circulation) and control of vital signs and its stabilization. In a conscious patient, the introduction is done simultaneously and permission is given to perform examinations, and in patients with a decrease in the level of consciousness, this is done for the patient’s family or companions)

- 2.a) *If vital signs are stable:*

It is important to pay attention to the patient’s medical condition and transfer him to a medical center for further diagnostic tests and treatment measures. Medical conditions should be monitored along the route, and life-threatening warning signs should always be considered. Behavioral recommendation in dealing with these patients is similar to the previous section.

- 2.b) *If vital signs are not stable*:

Attempts to stabilize the patient’s vital signs (based on the patient’s condition and needs, IV line implantation, opening the patient’s airway and starting serum therapy, etc.) should be considered and then the patient should be transferred to the nearest medical center as soon as possible. Behavioral and non-pharmacological interventions can be performed as much as possible depending on the patient’s medical and environmental conditions.

It is also important to gather evidence and document findings on the scene, any findings that help to understand the means of suicide (Like empty cans of pills and ropes, etc.) and evidence that is effective in understanding the cause of suicide (Patient’s will or any manuscript, patient medical prescriptions, patient medications, etc.) [44].

- C) *If the patient has died*

The first step is to ensure the definitive death of the patient. Call the police to be present at the scene, because, this may be murder instead of suicide. In the next step, it is important to pay attention to the emergency psychological needs of patients’ families. Usually, families in such situations are in a critical situation and may show impulsive reactions reactions [45]. Tips for dealing with such situations are provided in Table 4.

**Table 4:** Important points in dealing with committed suicide

1. Ask for help from police (who has been called at the beginning to provide security, he must record clues at the scene indicating the cause of death to report to forensics) 2. Do not manipulate evidence and documents in the scene) Avoid destroying police clues as number 1( 3. Do not remove the body of the deceased from the scene (For the same reason number 2) 4. Express empathy with the family and use verbal calming techniques 5. avoid judgmental behaviors with the family 6. In families with severe restlessness use medications (oral lorazepam 2 mg tablets) 7. Educate family about the condition and the normalcy of experiencing symptoms of acute psychological stress. Advise them to go to public-university clinics or call the crisis hotline for help from psychologists. 8. Encourage family to contact relatives and other people who can help them 9. Provide information and telephone number of centers that can be contacted, if they would need it. 10. If the situation does not improve with these initial measures, contact the dispatch center and get an assignment	

## Discussion

Several studies have been conducted on suicide management, as one of the most important issues in psychiatric emergencies. However, in most studies, more attention has been paid to the management of these patients in hospital emergency department than in the pre-hospital emergency department. In 2004, for example, the Australian National Institutes of Health provided a guidance for managing suicides in the emergency department, which was a good guide for emergency room therapists.

Lipton developed a clinical guideline of prevention, assessment, and management of suicide for pre-hospital technicians in 2005. However, drug management is not covered in this article [17]. Dr. Chunduri et al. used focus group discussions of psychiatrist working in emergency psychiatry wards to write a clinical guideline for assessment and management of suicide in 2017 [29].

Our purpose in writing this protocol was to increase the technician’s skills in the following areas:

1. Reducing the risk to himself, patient, patient’s family and those present at the scene.
2. Diagnosing the possible medical origins of the life-threatening medical symptoms and comorbidities
3. Quick assessment of the risk of imminent suicide
4. Establishing a therapeutic relationship with the patient and his family
5. Management of the suicidal patient and helping in management of the crisis
6. Collecting and recording the evidence related to suicide in the scene, both to assist hospital staff and to protect themselves from possible future legal problems.

Similar to the protocol for dealing with an acute psychiatric patient, the first step in this protocol is preparing the scene and ensuring security of the scene [16, 22].

The initial assessment and taking history in this protocol are the same as the general protocol for dealing with a psychiatric patient. However, it is especially important to ask about the history of any psychiatric disorder from the family of the suicidal subject. Assessment of risk factors (especially those that increase the risk of imminent suicide) as well as protective factors are the next crucial steps. However, according to emergency protocols in Iran, all of the suicidal patients must be transferred to a hospital, even if the technician considers the patient to be in a low risk for suicide. Although patients with low risk for suicide can be generally effectively cared for, monitored and followed-up at home, this decision is reserved for mental health professionals working in hospitals [30].

The protocol also emphasizes on assessment of risk factors for imminent suicide as opposed to chronic risk factors of suicide in the pre-hospital emergency setting due to time limitations [45, 46]. Furthermore, to reduce the negative reactions related to the stigma related to the word “suicide”, in this protocol we suggest technicians to ask patient about the decision and any immediate plan to harm oneself [21].

In the management of patients threatening suicide in our protocol, verbal intervention is the basis of treatment in line with other protocols [21, 47]. As in previous studies, it has been seen that many suicide threats are in fact a kind of protest against the conditions. Available and requesting a change and requesting assistance. Empathy and empathy for the patient’s condition and, if necessary, intervention in the crisis can be very helpful [47] Respect for the patient is very important and behaviors that induce the patient to be a failure or a sinner should be avoided. Attitudes that send the patient the message that “he does not know what is right?” And that he “does not have the authority to decide for himself” can also complicate matters [48].

Our protocol, like other protocols, prioritizes physical needs and risks in patients who have attempted suicide [42]. If behavioral interventions are not helpful, medications are considered [27]. Choice of medication and administration method are similar to that mentioned in the protocol for dealing with an acute psychiatric patient [16]. Additionally, although the main issue here is suicidality of the patient, the technician should address any other emergent symptoms such as aggression accordingly and not to ignore them as subsidiary [16].

This protocol, like others, has emphasized on paying attention to the emotional needs of the family of the patients who have committed suicide [27, 48, 49]. Feelings of anger or guilt towards the deceased is expected. Local religious and cultural teachings about the sinful act of suicide and the ambiguity of the fate of person who commits suicide also increase the pressure and stress of the family and mandates the intervention by technician as the first member of the treatment team to enter the scene.

## Conclusion

It is practical and useful to develop an evidence-based, but concise and portable protocol for pre-hospital emergency technician, which covers all of the major issues for a quick review before confronting a suicidal patient.

### Limitation

The number of articles and protocols on the management of suicide-threatening patients in the pre-hospital emergency, which we had access to, was limited. Most articles are related to the hospital emergency department. While the pre-hospital condition is very different.

### Recommendation

This protocol should be used in next researches and in emergency operations to identify weaknesses and operational glitches.

## Data Availability

The datasets during and/or analyzed during the current study available from the corresponding author on reasonable request.
